# 10-Jahres-Letalität, Krankheitsprogress und behandlungsassoziierte Nebenwirkungen bei Männern mit lokalisiertem Prostatakarzinom aus der randomisierten, kontrollierten ProtecT-Studie, analysiert nach erhaltener Therapie

**DOI:** 10.1007/s00066-021-01789-y

**Published:** 2021-05-07

**Authors:** Simon K. B. Spohn, Anca-Ligia Grosu

**Affiliations:** Universitätsklinikum Freiburg – Klinik für Strahlenheilkunde, Robert-Koch-Straße 3, 79106 Freiburg, Deutschland

## Hintergrund

2016 wurden in Großbritannien die Ergebnisse der multizentrischen und randomisierten Phase-III-ProtecT-Studie publiziert [1], die in einer großen Kohorte von Patienten mit primär lokalisiertem Prostatakarzinom (PCa) die Therapieoptionen des „active monitoring“ (AM) verglich mit den Ergebnissen nach radikaler Prostatektomie (RP) bzw. Strahlentherapie (RT). In dieser seinerzeitigen Intention-to-treat(ITT)-Analyse konnten keine signifikanten Unterschiede der PCa-spezifischen Letalität und der Letalität aufgrund anderer Ursachen zwischen den verschiedenen Therapieoptionen gezeigt werden. Dagegen waren der Krankheitsprogress und die Inzidenz von Metastasen in der AM-Gruppe signifikant höher als nach einer radikalen Therapie (RP oder RT). Männer, die der Studienteilnahme nicht zugestimmt hatten und ihre Behandlung selbst ausgewählt hatten, aber mit dem identischen Follow-up wie die Studienkohorte nachgesorgt wurden, nahmen diesmal im Rahmen einer ITT an der Auswertung teil. Deshalb publizierte die Arbeitsgruppe mit der hier kommentierten Arbeit eine Treatment-received-Analyse, an der, wie in der ProtecT-Studie beschrieben, sowohl Patienten der randomisierten Kohorte als auch Patienten, die über ihre Therapieoption selbst entschieden hatten, teilnahmen.

## Patienten und Methode

2664 Männer zwischen 50 und 69 Jahren, bei denen ein lokalisiertes PCa durch PSA-Screening diagnostiziert worden war, wurden in 9 Städten Großbritanniens in die Studie eingeschlossen. 1643 Männer willigten in eine Randomisierung ein, 997 Männer nicht. In der randomisierten Kohorte konnten 71 % der Patienten in der AM-Gruppe, 66 % in der RP-Gruppe und 66 % in der RT-Gruppe nach den D’Amico-Kriterien in ein Low-risk-Stadium (Gleason-Score = 6, PSA ≤ 10 µg/l, T‑Stadium ≤ cT2a) eingeteilt werden. In der Treatment-choice-Kohorte waren dies entsprechend 74 % in der AM-Gruppe, 61 % in der RP-Gruppe und 51 % in der RT-Gruppe. Patienten in der AM-Gruppe unterzogen sich im 1. Jahr 3‑monatlichen PSA-Kontrollen und anschließend 6‑ bis 12-monatlichen Kontrollen. Bei einem Anstieg von > 50 % in 12 Monaten wurde eine klinische Reevaluation durchgeführt. In der RP-Gruppe wurde bei Patienten mit einem PSA > 10 µg/l oder Gleason-Score ≥ 7 eine Lymphadenektomie und bei R1-Resektion oder extrakapsulärer Ausdehnung eine adjuvante Strahlentherapie durchgeführt. Patienten in der RT-Gruppe erhielten eine neoadjuvante Androgendeprivation (ADT) für 3–6 Monate vor und simultan zur Bestrahlung. Die Bestrahlung wurde in 3‑D-Technik mit 74 Gy in 37 Fraktionen durchgeführt.

Die PCa-spezifische Letalität war definiert als Todesursache, welche definitiv oder wahrscheinlich zumindest dem PCa zugeschrieben werden konnte. Eine metastasierte Erkrankung bezog sich auf Knochen-, viszerale und Lymphknotenmetastasen oder einen PSA-Wert > 10 µg/l. Progress wurde definiert als Nachweis einer Metastasierung, klinische T3/T4-Erkrankung, Langzeit-ADT, Ureterobstruktion, Rektalfistel oder Notwendigkeit eines Blasenkatheters wegen lokaler Tumorprogression.

Die Lebensqualität wurde mit „patient-reported outcomes“ (PROM) ermittelt, nämlich mit Expanded Prostate Index Composite (EPIC) und dem Fragebogen der International Continence Society Male Short Form (ICSmaleSF). Die dargestellte statistische Analyse umfasste die Treatment-received-Analyse der Randomised- und Treatment-choice-Kohorten. Zum Vergleich der Ergebnisse zwischen den Therapiegruppen wurde in beiden Kohorten ein „propensity score matching“ mit den Kovariaten cT-Stadium, Gleason-Score-Gruppe, logarithmisch-transformiertes PSA, Alter und Studienzentrum vorgenommen. In weiteren explorativen Analysen wurden die Daten beider Kohorten kombiniert.

## Ergebnisse

Von den 1643 Männern in der randomisierten-Kohorte erhielten 1260 (78 %) ihre zugewiesene Behandlung. In der Treatment-choice-Kohorte entschieden sich 507 (51 %) für AM, 262 (26 %) für RP und 189 (19 %) für RT (13 % für RT nach Protokoll und 6 % für eine andere RT inklusive Brachytherapie). Das mediane Monitoring der AM-Gruppe betrug 7,7 Jahre in der Randomised-Kohorte und 7,5 Jahre in der Treatment-choice-Kohorte. In letzterer unterschied sich die Wahl der Therapie deutlich und Patienten mit höhergradigem Risikoprofil entschieden sich häufiger für radikale Therapien („selection bias“).

In beiden Kohorten wurden in der AM-Gruppe mit ca. 45 % der Männer ähnliche Raten beobachtet, bei denen nach median 10 Jahren eine aktive Therapie durchgeführt wurde. Die ITT-Analyse mit „propensity score matching“ zeigte in beiden Kohorten der AM-Gruppe signifikant häufiger eine Metastasierung (6,0–6,1 % AM vs. 2,2–3,3 % RP vs. 1,7–3,2 % RT), ein Progress (17–24 % AM vs. 5,6–8,5 % RP vs. 6,3–8,6 % RT) und eine Initiierung einer ADT (6,5–8,8 % AM vs. 4,6–4,8 % RP vs. 5,3–6,2 % RT) bei allerdings geringen absoluten Differenzen. Die Treatment-received-Analyse erbrachte ähnliche Tendenzen, zeigte allerdings geringere Raten an Progress und einen späteren Beginn einer ADT in der AM-Gruppe der Treatment-choice-Kohorte.

Die gepoolte Analyse beider Kohorten erbrachte starke Hinweise darauf, dass RP oder RT, jeweils im Vergleich mit AM, die Inzidenz PCa-spezifischer Tode (Hazard Ratio [HR] 0,32–0,34), von Metastasierung (HR 0,33–0,40), Tumorprogress (HR 0,23) und Beginn einer ADT (HR 0,49–0,57) reduziert. Unterschiede bei Toden jeglicher Ursache konnten nicht ermittelt werden.

In der explorativen Analyse wurden die RP- und RT-Gruppen kombiniert und mit der AM-Gruppe verglichen. Aktive Therapie führte zu einer Reduktion der PCa-spezifischen Mortalität in beiden Kohorten (HR 0,34 in der randomisierten-Kohorte und 0,27 in der Treatment-choice-Kohorte, gepoolt 0,31 mit *p* = 0,003).

Die Analyse der PROM erbrachte 6 Jahre nach RP eine signifikant höhere Rate an Urininkontinenz (21 %) als nach AM (7,0 %) oder RT (2,3 %). In der RT-Gruppe bzw. RP-Gruppe berichteten 18,5 % bzw. 5 % der Patienten über eine erhaltene Potenz nach 6 Monaten. Im weiteren Verlauf erholte sich die Potenz geringfügig. Auch in der AM-Gruppe kam es zu einer kontinuierlichen Reduktion der Potenz, was das Alter und die Initiierung aktiver Therapien im Verlauf widerspiegelt.

## Schlussfolgerung der Autoren

Eine radikale Antitumortherapie des Low- und Intermediate-risk-PCa mit Prostatektomie oder Strahlentherapie reduziert die Rate an Metastasierungen oder Progress. Allerdings kann sie die sexuelle Potenz, die Urin- und die Darmfunktion beeinträchtigen. Die explorativen Analysen legen die Annahme nahe, dass radikale Therapien möglicherweise auch die PCa-spezifische Letalität senken bei insgesamt niedrigen Sterbezahlen. Bei der Bewertung dieser Ergebnisse müssen mögliche Bias- und Störfaktoren berücksichtigt werden.

## Kommentar

Das Prostatakarzinom ist nach wie vor die häufigste maligne Tumorerkrankung des Mannes. Aufgrund der unterschiedlichen Aggressivität der PCa steht den Ärzten und Patienten eine Vielzahl an therapeutischen Möglichkeiten zur Auswahl. Das „active monitoring“ soll v. a. bei Patienten mit Low-risk-Karzinomen eine Übertherapie vermeiden und nur Patienten mit klinisch relevanter Erkrankung einer kurativen Therapie zuführen. In dieser Patientenkohorte ist eine radikale Therapie insbesondere bei Patienten mit lokal begrenztem Prostatakarzinom und einer Lebenserwartung von mehr als 10 Jahren indiziert. Neben einer Reduktion der Sterblichkeit spielt hierbei auch die Verhinderung von Komplikationen infolge eines lokalen Tumorprogresses oder einer Metastasierung eine Rolle.

Die hier kommentierte ProtecT-Studie ist eine richtungsweisende Studie, deren 10-Jahres-Auswertung eine Äquivalenz von „active monitoring“, radikaler Prostatektomie und perkutaner Strahlentherapie hinsichtlich der PCa-spezifischen Letalität bei Patienten mit lokalisierter Erkrankung gezeigt hat [1]. Die langsame Tumorprogression des PCa mit hohem Risiko von den nicht-PCa-spezifischen Todesursachen zu unterscheiden, stellt eine besondere Herausforderung für das Design und die Interpretation von klinischen Studien dar, da hohe Patientenzahlen und lange Nachbeobachtungszeiträume von > 10 Jahren zur Evaluation des primären Endpunkts Gesamtüberleben notwendig sind. Die hier erfolgte Hinzunahme der 997 Patienten, die zwar nicht randomisiert, aber trotzdem studienkonform nachbeobachtet wurden, bot den Autoren die Möglichkeit, die Evidenz zu erhöhen. Diese Analyse erbringt zusätzliche Informationen als Basis für individuelle Therapieentscheidungen.

Die ITT- und die Treatment-received-Analysen der beiden Kohorten bestätigten die anfänglichen Studienergebnisse nach 10 Jahren mit einem geringen, aber signifikanten Vorteil der RP- und RT-Kollektive im Vergleich zum AM hinsichtlich der Metastasierung, der Tumorprogression und des Zeitintervalls bis zur notwendigen Initiierung einer ADT. Wegen der möglichen Komplikationen infolge der Metastasierung hat sich das metastasenfreie Überleben als validierter Surrogatparameter für das Gesamtüberleben etabliert und ermöglicht damit eine Verkürzung der üblicherweise mehr als eine Dekade dauernden Nachbeobachtungszeit [2].

Ähnlich den initialen Ergebnissen ist auch in der Treatment-choice-Kohorte die etwas geringere Rate an PCa-spezifischen Toden grenzwertig statistisch signifikant. In der explorativen, gepoolten Analyse beider Kohorten jedoch konnte ein signifikanter, aber in absoluten Zahlen weiterhin geringer Vorteil bezüglich des Gesamtüberlebens für radikale Therapien gezeigt werden. Eine dezidierte Auswertung nach Risikogruppen wurde zwar nicht explizit beschrieben, ist aber doch insbesondere im Hinblick auf das Intermediate-risk-Profil von Interesse. Die Daten zeigen nämlich im zusätzlichen Material, dass eine zielgerichtete Therapie bei Vorliegen eines höheren Gleason-Scores die Metastasierung und die PCa-spezifischen Tode verringert und übrigens auch die Indikation einer ADT verzögert. Diese Ergebnisse können im individuellen Fall als Basis dienen, eine radikale Therapie auch bei Low- oder Intermediate-risk-Profil zu rechtfertigen, insbesondere im jüngeren Erkrankungsalter oder weiterer hoher Lebenserwartung.

Zudem sind Fortschritte in der Diagnostik bei der Interpretation der Ergebnisse zu berücksichtigen. Während in der ProtecT-Studie der Gleason-Score mittels ultraschallgesteuerter transrektaler Biopsie ermittelt wurde, ist heutzutage die MRT-gesteuerte Biopsie weit verbreitet und ermöglicht eine bessere Detektion von Tumoren mit hohem Risikoprofil (Gleason-Score ≥ 4 + 3; [3]). Zudem ist die Genauigkeit von Biopsieergebnissen limitiert, ergibt die Histopathologie nach Prostatektomie doch in ca. 30 % der Fälle einen höheren Gleason-Score [4]. Dieser Aspekt ist bei der Hinzunahme der Treatment-choice-Kohorte nicht berücksichtigt und deshalb möglicherweise für die vorgestellten Ergebnisse mitursächlich. Fortschritte in der Diagnostik könnten in Zukunft diese Unsicherheit beseitigen. Viele Studien haben auch die Nützlichkeit von Radiomics-Analysen aus MRT-Daten zur Bestimmung der PCa-Aggressivität gezeigt [5]. Zudem hat sich die Positronenemissionstomographie mit Tracern gegen das prostataspezifische Membranantigen (PSMA-PET/CT) in der Diagnostik des PCa etabliert und liefert auch für die intraprostatische Tumordetektion wichtige komplementäre Informationen. Radiomics-Analysen der PSMA-PET/CT zeigen ebenfalls vielversprechende Ergebnisse zur nichtinvasiven Tumordetektion [6].

Bei der Therapieentscheidung sind insbesondere im Patientenkollektiv der Low- und Intermediate-risk-Patienten, wie oben bereits ausgeführt, neben dem onkologischen Outcome die Therapiefolgen zu bedenken. Die dargestellte Auswertung der PROM zeigte auch tatsächlich erhöhte Raten für Inkontinenz nach RP und ähnliche Raten für Nykturie in allen Therapiegruppen, erhöhte erektile Dysfunktionen insbesondere nach RP sowie häufiger Hämatochezie nach RT. Bei der Bewertung dieser Ergebnisse ist jedoch auf die überholten Therapietechniken der ProtecT-Studie hinzuweisen, deren Rekrutierungszeitraum 1999–2009 war. Der Großteil der Patienten (87 %) in der RP-Gruppe erhielt eine offene radikale Prostatektomie und die Patienten in der RT-Gruppe erhielten eine konventionelle 3‑D-Strahlentherapie. Eine prospektive Studie mit 2550 Patienten konnte in diesem Zusammenhang zeigen, dass unter Verwendung moderner Operations- (robotergestützte Op.) und Bestrahlungstechniken (intensitätsmodulierte Strahlentherapie [IMRT]) die RP nach 3 Jahren mit einer signifikanten Reduktion der sexuellen Dysfunktion und Urinkontinenz im Vergleich zu RT und AM einhergeht. Gastrointestinale Nebenwirkungen waren bei perkutaner RT häufiger als bei RP oder AM, zeigten aber nach 3 Jahren keinen signifikanten Unterschied [7]. Somit ist durch die heutigen technischen Möglichkeiten eine nebenwirkungsärmere Therapie als zum Zeitpunkt der Patientenrekrutierung der ProtecT-Studie zu erwarten. Neben einem exzellenten Nebenwirkungsprofil bietet die Strahlentherapie zudem mit der Brachytherapie und den modernen Fraktionierungsschemata zusätzliche Therapieoptionen. Die Evidenz der alleinigen High-dose-rate-Brachytherapie basiert in dieser Patientenkohorte größtenteils auf retrospektiven Studien. Allerdings zeigte eine prospektive monozentrische Studie mit 63 Patienten eine gute Verträglichkeit einer einmaligen HDR-Brachytherapie, deren Langzeitergebnisse bezüglich biochemischer Kontrolle allerdings noch ausstehen [8]. Des Weiteren verbreiten sich die moderat hypofraktionierte [9] und stereotaktische Strahlentherapie [10] insbesondere im angelsächsischen Raum, wodurch nichtinvasive, effektive und äquitoxische Therapieoptionen mit reduzierter Therapiedauer zur Verfügung stehen.

## Fazit

- Die hier besprochene Studie zeigt, dass kurativ ausgerichtete Therapien, wie Prostatektomie und Strahlentherapie, bei Low- und Intermediate-risk-PCa gleichwertig sind und den lokalen und distanten Progress verhindern können.
- Bei der Therapieentscheidung sind insbesondere im Patientenkollektiv der Low- und Intermediate-risk-Patienten neben dem onkologischen Outcome die Therapiefolgen zu bedenken. Die dargestellte Auswertung der PROM zeigte auch tatsächlich erhöhte Raten für Inkontinenz nach RP und ähnliche Raten für Nykturie in allen Therapiegruppen.
- Die Studie schafft Evidenz für eine höhere Letalität bei AM. Diese aktualisierten Daten können in dieser Patientenkohorte zur individuellen Therapieentscheidung herangezogen werden.
- Die Daten bieten im Kontext mit modernen Behandlungstechniken – operativ und radioonkologisch – und entsprechenden Therapieregimen eine effektive und sichere Behandlung.

*Simon Spohn und Anca-Ligia Grosu, Freiburg/Brsg.*
